# Functional mental capacity, treatment as usual and time: magnitude of change in secure hospital patients with major mental illness

**DOI:** 10.1186/s13104-015-1547-4

**Published:** 2015-10-14

**Authors:** Julieanne Dornan, Miriam Kennedy, Jackie Garland, Emer Rutledge, Harry G. Kennedy

**Affiliations:** National Forensic Mental Health Service, Central Mental Hospital, Dundrum, Dublin 14, Ireland; St Patricks Hospital, Dublin, Ireland; Department of Psychiatry, Trinity College Dublin, Dublin, Ireland

**Keywords:** Capacity, Consent, Mental illness, Time, Recovery

## Abstract

**Background:**

Decision making ability can change with time, depending on mental or physical health. Little is known about the factors that determine this change and the relationship of capacity to time. As a pilot for studies using functional mental capacities as an outcome measure, we sought to quantify this relationship measuring change over time using competence assessment tools, and rating scales for symptoms and global function.

**Methodology:**

We assessed 37 inpatients in a secure psychiatric hospital. All patients met the diagnostic and statistical manual of mental disorders-fourth edition and International classification of diseases, 10th edition criteria for an Axis 1 mental illness, all with psychosis. Patients were interviewed twice a mean of 323 days apart (median 176 days range 17–1221 days). The MacArthur competence assessment tools for consent to treatment (MacCAT-T) and fitness to plead (MacCAT-FP) were used to quantify functional capacity along with the Positive and Negative Syndrome Scale (PANSS) and global assessment of function (GAF) scale. A comparison was also made between those patients prescribed clozapine in comparison to other antipsychotics.

**Results:**

The number judged by treating psychiatrists to lack capacity either to make a treatment choice or to plead in court fell from 35 to 8 %. Change was greatest for those admitted within the previous 9 months. The measures of capacity improved between time 1 and time 2 for both consent to treatment and fitness to plead. The measures of capacity improved with positive symptoms within the PANSS and with GAF scores. Those with shorter lengths of stay at baseline had the greatest improvements in the MacCAT-FP scores. Effect sizes were medium or large (0.3–0.7+). As expected, patients prescribed clozapine had larger changes in functional mental capacities and larger effect sizes than those prescribed other psychotropics. The results show a strong relationship between the clinicians’ assessment of capacity and structured rating scales.

**Conclusions:**

We have shown that there is an improvement in capacity scores with time. More research is needed to compare the effect of treatment on capacity at fixed time intervals. It would also be helpful to look at a more general patient population.

## Background

It can be shown that modern psychiatric treatments help to restore functional mental capacities, even in patients with severe mental illnesses such as schizophrenia [1]. The extent to which functional mental capacities change over time is of great importance when considering the legal and human rights protections necessary for mentally incapacitated patients, including those detained and treated under mental health legislation. The time course of impairment and recovery, the extent to which impairments are short term or enduring, and the time scale over which changes can be expected, are seldom studied.

### Rationale

Research in schizophrenia and other severe and enduring mental illnesses should concentrate on functional outcomes [2]. Neurocognitive and social cognitive deficits underpin many of the most handicapping functional impairments in schizophrenia and schizoaffective disorder [3]. Impairment of mental capacity is a particularly serious manifestation of mental disorder, leading to impairment of the ability to live independently, to exercise one’s rights and to function in society. A functional approach is considered best practice for the assessment of mental capacity in legal contexts [4]. The respect for an individual’s capacity to refuse or consent to treatment is an essential aspect of the individual’s human rights. Capacity itself is dynamic and can change with time. It is essential that research in this area is conducted to facilitate further knowledge and ensure best practice.

Separate functional mental capacities such as ability to give or withhold consent to treatment and fitness to stand trial are not independent of each other [5]. In addition the amount of information about treatment can directly affect the capacity of a person to make a choice [6]. As clinicians we strive to protect patients’ interests including the right to self-determination, respect for autonomy and the principle of reciprocity by providing information about treatments in order to restore or optimise autonomy [7]. Clinical judgement must be exercised concerning the amount of information disclosed but deciding what is material to the individual is difficult when few items of information can be processed. Clarifying the extent and the limits of functional mental capacity would help when deciding the thresholds at which the use of assisted decision making, guardianship and independent second opinions should be deployed to ensure the protection of the human rights of impaired persons. Knowledge of the likely time course for changes in mental capacity in the course of treatment for severe, enduring and disabling mental illnesses is necessary if a recovery orientation is to be meaningful.

Based on our experience of using functional mental capacity as a meaningful outcome measure for a treatment intervention [1], we sought to examine the parameters which might influence susceptibility to change in functional mental capacity in future treatment studies. We considered factors such as length of stay and the interval between measurements. We also sought to assess the likely effect size for an intervention that might produce a change in measures of functional mental capacity, such as clozapine for treatment resistant psychosis.

### Objectives

In this pilot study, in order to provide an evidence base for future studies using functional mental capacities as outcome measures, we set out to assess the extent to which mental capacities change over time, the basic parameters influencing change and the likely effect sizes. We hypothesised that factors influencing change in functional mental capacities over time would include time since admission and the length of the interval between measurements. We also hypothesised that changes in symptom severity and global function would be associated with changes in functional mental capacity measures. We therefore measured the functional mental capacity of patients in a secure forensic hospital at different times after admission and change over a wide range of time intervals. In this exploratory study we also wished to assess the expected effect size for changes in measures of functional mental capacity before and after a period of observation or treatment as usual.

## Methods

### Sample and setting

This study sample consisted of 37 patients admitted to a secure forensic hospital, the Central Mental Hospital, Dundrum, part of the National Forensic Mental Health Service for Ireland. We have previously described the role of this forensic hospital [8], which is similar to the role of forensic hospitals in other jurisdictions. All patients are detained under mental health legislation, the majority while on remand pending trial or following trial.

During the period of this study, ‘treatment as usual’ consisted of a target of 25 h of programmed activities a week for each patient as part of an individualized care and treatment plan. Care and treatment was organized into a system of ‘pillars of care’ including programmes for physical health, mental health, substance misuse, problem behaviours, self-care and activities of daily living, education occupation and creativity, and family and social networks [9].

Patients met the ICD-10 criteria (World Health Organisation, 1993) [10] and DSM-IV –TR criteria (American Psychiatric Association, 2000) for an Axis 1 mental disorder [11].

Research participants were interviewed on two occasions, at baseline “Time 1” and at follow up “Time 2”. All patients remained inpatients in the Central Mental Hospital during the study period.

The patient’s length of stay in hospital was recorded at each interview. Assessment times were chosen randomly to ensure patients were assessed at varying stages of admission, treatment and recovery. Information pertaining to the patient’s pharmacological treatment was recorded.

The study was approved by the Central Mental Hospital research, audit and effectiveness committee. All subjects gave informed consent to participate in the study. An assessment of functional mental capacity to give consent to the research interviews was not required as part of the consent process by the research, audit and effectiveness committee. The committee considered that Articles 5, 6 and 17 of the Council of Europe Convention on Human Rights and Biomedicine were fully complied with (the Orviedo Convention) [12] and Article 6 of the Additional Protocol concerning capacity to consent [13].

### Reliability

Patients were interviewed by post-membership psychiatrists, trained in the use of the research instruments. Inter-rater reliability was assessed by joint interviewing to yield a Cohen’s kappa value for the two McArthur instruments of greater than 0.946.

### Exclusion criteria

Because this was intended as a study of functional mental capacity in patients with severe mental illnesses, exclusion criteria included a primary diagnosis of delirium, dementia or learning disability (moderate/severe) or other cognitive disorders or inadequate understanding of the English language.

### Measurement tools

The Macarthur competence assessment tool-consent to treatment [14, 15] and the Macarthur competence assessment tool-fitness to plead (MacCAT-FP) [16] were used. These are well validated structured interview rating scales for the measurement of functional mental capacities. All the participants were inpatients detained by the criminal courts and all were subject to mental health legislation. Decisions regarding competence to consent to treatment and competence to stand trial were therefore relevant to each patient.

The MacCAT–T measures understanding, reasoning and appreciation in relation to a proposed treatment, and the ability to communicate a choice [14, 15]. It has also been validated in England [17]. Each patient is given individual information on their disorder, including symptoms and diagnosis. They are then informed about the nature, benefits and risks of the three treatment options. In this study the decision scenario involved an oral antipsychotic, a second oral antipsychotic or no medication. For each of the 3 options, 2 positive and 2 negative pieces of information were given. There were 12 items of information in total. The framing of information regarding risk uses both verbal and numerical information [5, 6].

‘Understanding’ examines the patient’s ability to retain and retell information in their own words. ‘Reasoning’ assesses the ability to foresee the consequences of their choices and to make comparisons among various options. ‘Appreciation’ rates the ability of the patient to personalise to oneself the information given concerning health and the potential benefits of treatment. Expressing a choice is a simple binary rating.

The Positive and Negative Symptom Scale (PANSS) was used as a validated measure of symptom severity [18].

The global assessment of function (GAF) was used as a measure of ‘real world’ ability to function [19]. This was rated by the researchers who consulted with the primary nurse and key worker of each patient.

### Criterion and outcome measures

Each patient was clinically assessed by their treating psychiatrist to determine functional capacity in two domains—capacity to consent to treatment and fitness to plead. These ratings were made blind to the research assessment of functional capacities. These two assessments were taken as criterion measures because they would be accepted in legal settings.

The sample was divided into 2 groups—one group received clozapine (n = 7) treatment and the second group (n = 30) received other psychotropic medication.

### Statistics

Statistics were calculated using SPSS-20 [20]. The distributions of the group scores were tested for statistically significant differences.

Paired T-tests were used to compare changes in mean scores from T1 to T2.

The Pearson correlation coefficient (r) was used to measure the strength and direction of the relationship between pairs of variables. We included all difference score variables for MacCAT-T, MacCAT-FP, PANSS and GAF.

A one way between group analysis of variance was conducted to explore the effect of a pharmacological treatment on changes in measures of functional capacity between Time 1 and Time 2.

Cohen’s d and its 95 % confidence interval was calculated as a measure of effect size in relation to change over time and when comparing change between those treated with clozapine and others. This was calculated to facilitate power calculations for future studies using these measures of functional capacity as outcome measures.

## Results

Out of 37 patients interviewed, 34 (91.8 %) were male and 3 (8.2 %) were female. The mean age was 32.3 years (age range 19.8–56.4 years) at initial interview (Time 1) and 33.2 years (age range 19.9–56.8 years) at follow up interview (Time 2). All patients met the ICD-10 criteria and DSM-IV-TR criteria for schizophrenia (n = 31), schizoaffective disorder (n = 2), bipolar affective disorder (n = 2), severe depressive episode with psychotic symptoms/ major depressive disorder with psychotic features (n = 1) and psychotic disorder due to use of psychoactive substances/ substance induced psychotic disorder (n = 1).

The mean length of stay at Time 1 was 815 days (length of stay range 1–6265 days, median 275 days) and at Time 2 was 1162 days (length of stay range 35–6422 days). The mean time interval between Time 1 and Time 2 was 323 days (Time 1–Time 2 interval range 17–1221 days, median 176 days).

When corrected for multiple testing, neither the length of stay at T1 nor the interval between T1 and T2 correlated with any measure of change in the MAC-CAT-T or MAC-CAT-FP scores or sub-scales, changes in the PANSS score or sub-scales or the GAF. However, when only those with a length of stay at T1 less than the median were chosen (less than 275 days, n = 18), a negative correlation emerged between length of stay at T1 and change in the MacCAT-FP between T1 and T2 (MacCAT-FP understanding r = −0.565, p = 0.015, reasoning r = −0.710, p = 0.001, appreciation r = −0.537, p = 0.022, total score r = −0.647, p = 0.004) so that the shorter the length of stay at T1, the greater the subsequent increase (improvement) in score. No other associations were found with MacCAT-T, PANSS or GAF.

### Criterion measures

At the time of assessment 1 (Time 1) 8 (21.6 %) of the subjects were rated unfit to plead by their treating psychiatrist and 9 (24.3 %) were rated unable to make a choice in relation to treatment; At Time 1, 24 (64.8 %) were competent by both criterion tests; 13 (35.1 %) were either unfit to plead or did not express a treatment choice or both. The two criterion tests of functional mental capacity were not significantly associated at Time 1 (X^2^ = 3.7, df = 1, p = 0.56).

At follow up assessment (Time 2), 2 (5.4 %) of subjects were rated by their treating psychiatrist as unfit to plead and 2 (5.4 %) were rated not capable of making a choice in relation to treatment. At Time 2, 34 (91.9 %) were competent by both criterion tests and 3 (8.1 %) were either unfit to plead or did not express a treatment choice. The two criterion tests were then associated (X^2^ = 8.2, df = 1, p = 0.004) (Tables 1, 2, 3, 4, 5).

**Table 1:** Fitness to plead and ability to make a choice rated as per treating Psychiatrist at time 1 and 2

	Time 1, n (%)	Time 2, n (%)
Fitness to plead		
Fit to plead	29 (78.3)	35 (94.6)
Unfit to plead	8 (21.6)	2 (5.4)
Ability to express a treatment choice		
Expresses a treatment choice	28 (75.7)	35 (94.6)
Cannot express a treatment choice	9 (24.3)	2 (5.4)
Either unfit or unable to express a treatment choice		
Capable (both)	24 (64.8)	34 (91.9)
Either incapacity	13 (35.1)	3 (8.1)

**Table 2:** Paired t test evaluating differences in mean scores of functional capacity, GAF and PANSS at time 1 and 2

	Time 1		Time 2		Time 2-Time 1			Effect size			ANOVA	
	Mean	SD	Mean	SD	Mean difference	95 % CI		Cohen’s d	95 % confidence interval		T	p
	N = 37		N = 37		N = 37	Lower	Upper		Lower	Upper	df = 36	2-tailed
MacCAT-T												
Understanding	4.1	1.8	5.1	1.4	0.9	0.4	1.5	0.62	0.15	1.09	3.6	0.001
Reasoning	3.0	2.6	4.7	2.8	1.6	0.5	2.7	0.63	0.16	1.09	3.0	0.004
Appreciation	1.7	1.6	2.3	1.5	0.7	0.2	1.1	0.39	−0.07	0.85	3.2	0.003
Total	8.9	5.1	12.1	5.0	3.2	1.6	4.8	0.63	0.17	1.10	4.1	<0.001
MacCAT-FP												
Understanding	10.9	5.0	12.5	4.4	1.6	2.2	3.0	0.34	−0.12	0.79	2.3	0.025
Reasoning	6.5	3.8	7.7	3.4	1.2	0.1	2.4	0.33	−0.13	0.79	2.2	0.038
Appreciation	7.3	4.5	8.6	3.8	1.2	0.1	2.6	0.31	−0.14	0.77	1.8	0.075
Total	24.8	12.5	28.8	10.4	4.1	0.5	7.6	0.54	0.07	1.00	2.3	0.025
GAF	49.8	16.5	59.7	16.0	9.8	4.2	15.5	0.61	0.14	1.08	3.6	0.001
PANSS												
PANSS positive	15.7	8.1	12.2	5.7	−3.6	−5.7	−1.5	0.49	0.04	0.96	−3.5	0.001
PANSS negative	20.1	6.9	17.4	7.9	−2.7	−5.5	0.1	0.38	−0.07	0.84	−1.9	0.058
PANSS general	34.5	7.9	27.4	8.0	−7.1	−9.7	−4.4	0.89	0.42	1.37	−5.4	<0.001
PANSS total	70.4	17.3	56.6	19.5	−13.8	−19.8	−7.7	0.75	0.28	1.22	−4.7	<0.001

**Table 3:** For those who changed from unfit to plead to fit as judged by their treating psychiatrist, one way ANOVA of change in measures of functional capacity between Time 1 and Time 2

Mean (standard deviation)	No change in clinicians’ assessment of fitness to plead, n = 31		Change in clinicians’ assessment of fitness to plead, n = 6		Effect size			ANOVA	
	Mean	SD	mean	SD	Cohen’s d	95 % confidence interval		F	P
	N = 31		N = 6			Lower	Upper		
MacCAT-T difference T2 − T1									
Understanding difference	0.61	1.3	2.7	1.7	1.38	0.45	2.31	10.8	0.002
Reasoning difference	1.62	3.1	1.5	4.0	0.03	−0.84	0.91	0.0	0.930
Appreciation difference	0.64	1.3	0.75	1.4	0.08	−0.79	0.96	0.0	0.857
Total difference	2.9	4.4	4.9	6.4	0.36	−0.51	1.24	0.9	0.344
MacCAT-FP difference T2 − T1									
Understanding difference	0.83	3.6	5.7	4.9	1.13	0.22	2.04	7.9	0.008
Reasoning difference	0.58	3.1	4.5	3.4	1.20	0.29	2.12	7.9	0.008
Appreciation difference	0.42	3.5	5.50	5.0	1.17	0.26	2.09	9.4	0.004
Total difference	1.80	8.8	15.7	12.1	1.31	0.39	2.24	11.1	0.002

Note that six who had been unfit became fit by T2

**Table 4:** For those who changed from unable to express a treatment choice to able, one way ANOVA of change in scores on McCAT-T and MacCAT-FP (T2 − T1)

Means (standard deviation)	No change in ability to express a treatment choice		Change in ability to express a treatment choice		Effect size			ANOVA	
	Mean	SD	Mean	SD	Cohen’s d	95 % confidence interval		F	P
	N = 30		N = 7			Lower	Upper		
MacCAT-T difference T2 − T1									
Understanding difference	0.6	1.4	2.4	1.7	1.16	0.29	2.02	7.7	0.009
Reasoning difference	1.3	3.4	2.7	2.2	0.49	−0.34	1.32	1.0	0.32
Appreciation difference	0.5	1.3	1.1	1.2	0.48	−0.36	1.29	1.2	0.27
Total difference	2.5	4.8	6.2	3.6	0.87	0.03	1.72	3.5	0.68
MacCAT- FP difference T2 − T1									
Understanding difference	0.87	3.7	4.9	4.9	0.93	0.08	1.78	5.8	0.022
Reasoning difference	0.6	3.0	3.8	3.9	0.92	0.07	1.77	5.8	0.021
Appreciation difference	0.27	3.1	5.4	5.3	1.18	0.32	2.05	11.5	0.002
Total difference	1.7	8.7	14.1	12.4	1.16	0.29	2.02	9.8	0.003

Note that 7 who had been unable to express a choice became capable

**Table 5:** Change in scores for MacCAT-T between T1 and T2 comparing those treated with clozapine (n = 7) and all others (n = 30)

Mean (standard deviation)	Other psychotropics	Clozapine	Effect size			ANOVA	
			Cohen’s d	95 % confidence interval		F	P
	N = 30	N = 7		Lower	Upper		
MacCAT-T difference T2 − T1							
Understanding difference	0.7 (1.4)	2.0 (2.0)	0.75	−0.09	1.59	4.1	0.051
Reasoning difference	1.2 (3.1)	3.4 (3.1)	0.71	−0.13	1.55	2.9	0.097
Appreciation difference	0.3 (1.0)	2.3 (0.9)	2.10	1.15	3.05	22.0	0.001
Total difference	2.2 (4.2)	7.7 (4.8)	1.22	0.35	2.09	9.3	0.004

A paired sample T test was conducted to evaluate the differences in mean scores on tests of functional capacity, GAF and PANSS at Time 1 and Time 2. Table 2 shows that the overall MacCAT-T measure improved between Time 1 and Time 2. Measures of understanding, reasoning and appreciation all improved with time. All results were statistically significant. The MacCAT-FP total and individual measures of capacity also improved significantly with time with the exception of the appreciation scores which improved marginally.

The PANSS total and subscale scores fell (improved) between Time 1 and Time 2. Significantly lower PANSS positive symptom and general symptom scores were observed at Time 2 compared to Time 1 with a marginal improvement in PANSS negative symptom scores.

For the GAF, significantly higher (better) mean scores were observed at Time 2.

### Correlations

The relationship between changes in scores for the variables (T2 − T1 = change) were tested. There was an inverse relationship between MacCAT-FP-Total ‘change’ and PANSS Positive ‘change’ r = −0.358 (p = 0.030). MacCAT-FP-Appreciation ‘change’ and PANSS Positive ‘change’ r = −0.366 (p = 0.026). This shows an association between a decrease in psychopathology and an improvement in some measures of capacity over time. The correlations between PANSS positive change and MacCAT-FP understanding ‘change’ (r = −0.285) and reasoning ‘change’ (r = −0.309) did not reach significance.

All changes in PANSS sub-scores correlated inversely with changes in GAF scores: PANSS positive ‘difference’ and GAF ‘difference’ r = −0.547 (p < 0.001), PANSS negative ‘difference’ and GAF difference r = −0.424 (p = 0.009), PANSS general ‘difference’ and GAF ‘difference’ r = −0.550 (p = 0.000), PANSS-total ‘difference’ and GAF ‘difference’ r = −0.633 (p < 0.001). This shows that with a reduction in psychopathology there is an improvement in the global level of functioning between Time 1 and Time 2.

A one way between group analysis of variance was conducted to explore the association between the treating clinician’s assessment of fitness to plead and changes in measures of functional capacity between Time 1 and Time 2. The sample was divided into 2 groups—one group for whom the clinicians’ assessment of fitness to plead changed (all from unfit to fit n = 6) between Time 1 and Time 2 and the second group (n = 31) for whom the clinicians’ assessment of their fitness to plead did not change between Time 1 and Time 2. There was a significant difference at the p < 0.05 level in the mean change in MacCAT-T- understanding and MacCAT-FP understanding, reasoning, appreciation and total scores for the 2 groups.

### Change in capacity to make a treatment choice

A one way between group analysis of variance was conducted to compare those for whom the treating psychiatrist found had regained the ability to make a treatment choice (n = 7, all became competent) with those who had no change in ability to make a treatment choice (n = 30). Changes in measures of functional capacity between T1 and T2 were compared. There was a significant difference at the p < 0.05 level in the mean change in MacCAT-T- understanding and in MacCAT-FP understanding, reasoning, appreciation and total scores for the 2 groups.

### Clozapine

There was a significant difference at the p < 0.05 level in the mean change in MacCAT-T- Total and MacCAT-T appreciation scores for the 2 groups.

## Discussion

### Key results

We have shown that measures of functional capacity can change over time while receiving ‘treatment as usual’ in a secure forensic hospital. We found improvements between Time 1 and Time 2 for both capacity to consent to treatment and fitness to plead.

Improvement in measures of functional capacity occurred for total scores and sub-scale scores. This was also evident in the clinical assessment of capacity. Improvements in functional mental capacity were accompanied by improvements in symptom severity and in global function. The magnitude of change was greatest when assessment commenced within the first 9 months (275 days) after admission though this was statistically significant only for the MacCAT-FP. The length of the interval between assessments did not appear to relate to the magnitude of change. Clozapine treatment appeared to be associated with greater improvements in functional capacity scores. Effect sizes generally were moderate (greater than 0.3) or large (greater than 0.7).

### Limitations

This study has many drawbacks. The patients are highly selected forensic patients and so these results may not generalise to other patient groups. Patients with schizophrenia and schizoaffective disorder accounted for 89 % of the sample with the remainder made up of affective psychoses. The interval between T1 and T2 had a very wide range, although this was also an advantage when exploring an unquantified phenomenon. It is possible that for some patients, measures of functional mental capacity might have varied more than once (improved, deteriorated, improved again) during the longer observation periods.

The numbers assessed are relatively few. A larger sample would have permitted further analysis of interactions between factors influencing change in mental capacities. Complex inter-relationships are likely between symptom severity, global function and functional mental capacities. A much larger sample would be required in order to study such effects.

The clinical assessment of capacity to make a treatment choice and fitness to plead agreed much more closely at the end of the study than at the beginning. This may indicate a true difference between thresholds for these criteria, or greater consistency about competence (more common at the end of the study) than about incompetence (more common at the beginning of the study), as appears to have been the case.

### Interpretation

The clinical factors relevant to change in functional capacity can only be described tentatively based on this study. It appears that largest effect sizes for change in functional mental capacities occur when measurement commences during the first year after admission. Measurements of functional mental capacity and global function improved at the same time as improvement in PANSS positive symptoms such as active delusions, hallucinatory behaviour and excitement. There were no statistically significant relationships between changes in measures of functional mental capacity and changes in negative symptoms such as disturbance of volition, active social avoidance and poor attention. The measurement of global function was also associated with improvement in all measures of mental capacity. Other researchers have shown complex relationships between insight (awareness of illness), symptoms and executive function, with different measures improving at different time intervals in the year after first presentation with schizophrenia spectrum disorders [21]. Psychiatric and general hospital patients may differ according to whether impairments of understanding, reasoning or appreciation are the key elements impairing decision making capacity [22].

Studies of change in mental capacity over time, and studies relating this to treatment, remain rare [1]. The MacArthur competence assessment tool-fitness to plead (MacCAT-FP) has been shown to be sensitive to change, with improvements in capacity during hospital treatment [16]. In one recent 18 month longitudinal study concerning patients with schizophrenia, 20 % improved and 24 % had worsening capacity to give or withhold consent to research, with 4 % falling below a pre-determined threshold over the course of the study [23]. A longitudinal study of patients with dementia over 9 months using the MacCAT-T also demonstrated the expected decline in decision making capacity, particularly reasoning, and in neuropsychological function including logical memory [24]. A further study using the MacArthur Consent to Research instrument showed modest time dependent improvements in understanding and reasoning with no change in appreciation scores and general stability. There was more variation in scores for patients with schizophrenia compared to bi-polar disorder [25].

The treating psychiatrist is responsible for the prescription of psychotropic medication in alleviating the symptoms of psychotic symptoms. In this study several antipsychotics were prescribed. A comparison was made between those patients who were prescribed clozapine and those who were prescribed other antipsychotic medications. Results show that those who were prescribed clozapine had greater improvements in some measurements of capacity than those prescribed other psychotropic medications.

Treatment included much more than medication. All patients had an individual care plan addressing physical health, mental health, substance misuse problems, problem behaviours, activities of daily living, education and occupation, family and other relationships [9, 26]. A minimum quality standard aims for 25 h per week of meaningful activity composed within these ‘pillars’ of care. This represents ‘treatment as usual’ in this forensic hospital at this time. When treatments are individualised and complex, time since admission and time between assessments may emerge as the only correlates of treatment that can be aggregated and analysed. However we have published the results of specific assessment instruments for progress in these domains as related to leave, moves between levels of therapeutic security and conditional discharge [27–29]. We have also published results of assessments of neurocognitive and social cognitive impairments in patients with schizophrenia and schizoaffective disorder in a forensic secure hospital [3]. Future research in this group of patients will study the relationship between measures of neurocognitive and social cognitive function, treatment participation, engagement and changes in functional mental capacity.

Other factors that might have influenced the improvement in capacity scores over time while in a secure forensic hospital might include abstinence from alcohol and illicit substances and a benzodiazepine free environment. Hospital care also provides a safe, structured environment with daily activities. This may diminish the stress and arousal associated with mental illness and the associated socioeconomic deprivation, relationship problems, high expressed emotion and chaotic lifestyle.

### Generalisability

Although we have demonstrated an improvement in capacity scores over time, we cannot definitively say what this improvement is attributed to. Further research assessing mental capacities and other variables as soon as possible after admission and at fixed intervals subsequently, up to a year or more would be required to establish time courses and causal relationships between treatments, symptoms, global function and functional mental capacities. Research is also needed in a non-forensic population of psychiatric patients.

In this sample of patients with severe, enduring and disabling mental disorders, these results indicate that treatment given to alleviate symptoms and restore a person’s capacity is broadly effective. This is in line with the ethical principle of reciprocity—if a person’s autonomy is limited by law and their decisions are made by substitution, then treatment should offer at least the possibility of restoring decision making capacities [4]. In addition, there is always an assumption of capacity. Capacity to consent to research is a functional capacity. Like all functional capacities it is specific to that function. A person may lack capacity to consent to treatment but retain another functional capacity e.g. to enter a plea in court, or to make a will. Therefore evidence that a person or members of a study sample lack functional capacity to consent to treatment is not evidence that they lack capacity to consent to research. In the present study, we found that the two criterion measures of functional capacity – to consent to treatment and to plead in court—were not correlated at baseline. It follows that there is no evidence for an assumption of impaired capacity to consent to research at baseline.

## Conclusions

The aim of the study was to assess the relationship between functional mental capacities and time. It has been demonstrated that there is an improvement in capacity scores with time. This is greatest in the first year after admission. The results of this study show good agreement between the clinician’s assessment of capacity and the structured rating scales. This reinforces the importance of combining clinical judgement with semi structured tools for assessment of capacity [14–16, 30]. The Macarthur competence assessment tools are suitable instruments for assisting the clinician in detecting deficits in decision making abilities. These instruments provide a structured way of assessing capacity, or of assisting the clinician in assessing capacity, both when considering that capacity might be impaired and when considering whether capacity has been restored.

They should always be used as part of a thorough clinical evaluation to ensure that other factors that might limit decision making autonomy are also assessed.

Translating the research validation of structured professional judgement instruments such as these into routine practice would be facilitated by clinical training standards and by legislative structures protecting the rights of the mentally impaired or incapacitated. The use of such instruments may be worth considering when setting standards for expert evidence and when training judges or tribunals in assessing the quality of expert evidence.

## Abbreviations

ICD-10: International classification of diseases, 10th edition
DSM-IV: the diagnostic and statistical manual of mental disorders-fourth addition
MacCat-T: Macarthur competence assessment tool-consent to treatment
MacCat-FP: Macarthur competence assessment tool-fitness to plead
PANSS: Positive and Negative Symptom Scale
GAF: global assessment of functioning
